# Development of Lomustine and *n*-Propyl Gallate Co-Encapsulated Liposomes for Targeting Glioblastoma Multiforme via Intranasal Administration

**DOI:** 10.3390/pharmaceutics14030631

**Published:** 2022-03-12

**Authors:** Gábor Katona, Fakhara Sabir, Bence Sipos, Muhammad Naveed, Zsuzsanna Schelz, István Zupkó, Ildikó Csóka

**Affiliations:** 1 Institute of Pharmaceutical Technology and Regulatory Affairs, Faculty of Pharmacy, University of Szeged, Eötvös Str. 6, H-6720 Szeged, Hungary; fakhra.sabir@gmail.com (F.S.); sipos.bence@szte.hu (B.S.); csoka.ildiko@szte.hu (I.C.); 2 Department of Pharmacology and Pharmacotherapy, Faculty of Medicine, University of Szeged, Dóm Sqr. 12, H-6720 Szeged, Hungary; naveed.muhammad@med.u-szeged.hu; 3 Department of Pharmacodynamics and Biopharmacy, Faculty of Pharmacy, University of Szeged, Eötvös Str. 6, H-6720 Szeged, Hungary; schelz.zsuzsanna@szte.hu (Z.S.); zupko@pharm.u-szeged.hu (I.Z.)

**Keywords:** lomustine, *n*-propyl gallate, glioblastoma multiforme, liposome, nasal delivery, controlled release

## Abstract

This work aimed to develop lomustine (LOM) and *n*-propyl gallate (PG)-loaded liposomes suitable for targeting glioblastoma multiforme (GBM) via the auspicious nose-to-brain drug delivery pathway. The therapeutical effect of LOM, as a nitrosourea compound, can be potentiated by PG suitable for enhanced anti-cancer therapy. Nose-to-brain delivery of PG and LOM combined in liposomes can overcome the poor water solubility, absorption properties, and toxicity issues in the systemic circulation. Optimization and characterization of the liposomal carrier with binary drug contents were carried out in order to achieve adequate encapsulation efficiency, loading capacity, drug release, and ex vivo permeation. The optimized liposome co-encapsulated with both drugs showed suitable Z-average (127 ± 6.9 nm), size distribution (polydispersity index of 0.142 ± 0.009), zeta potential (−34 ± 1.7 mV), and high encapsulation efficacy (63.57 ± 1.3% of PG and 73.45 ± 2.2% of LOM, respectively) meeting the acceptance criteria of nose-to-brain transport for both drugs. MTT assays of PG-LOM formulations were also conducted on NIH/3T3 (murine embryonic fibroblast), U87 (glioblastoma), and A2780 (ovarian cancer) cell lines indicating reduced an antiproliferative effect on all types of cells. Our results supported the use of this novel combination of LOM and PG in a liposomal formulation as a promising carrier for glioblastoma targeting via the intranasal route.

## 1. Introduction

Glioblastoma multiforme (GBM) is an aggressive and the most recurrent form of the central nervous system (CNS) tumors, which accounts for 65% of the cases of brain tissue-associated tumors. The poor prognosis, uncontrolled cell division, diffuse infiltration, and significant angiogenesis are associated with the main GBM characteristics [1,2]. The effective treatment for GBM belongs to unmet medical needs. The morphologically diffuse and heterogeneous nature of GBM makes it especially challenging for therapeutics to access the tumor site [3]. Besides the structural limitations, the blood–brain barrier (BBB) forms another obstacle limiting the successful delivery of antineoplastic agents to target GBM, which is the major drawback of therapy through conventional administration routes. To overcome these obstacles, novel strategies and drug carriers are required to enhance the successful localization of chemotherapeutics to the tumor site [4].

To deliver chemotherapeutics to the target site, the common treatment strategy is to apply them peripherally (parenteral or oral), however, these delivery routes reduce the potency of the drug molecule leading to low brain targeting efficacy [5]. Moreover, the first-pass effect, systemic clearance, enzymatic degradation, plasma protein binding, and the volume of distribution can reduce the bioavailability of drugs through conventional administration routes [6]. Intranasal administration (IN) can overcome the limitations of conventional drug delivery and allows a faster delivery process [7]. Nose-to-brain delivery is a simple, direct approach resulting in a shorter onset of action, improved targetability, low systemic toxicity, and clearance [8]. In addition to that, an increased concentration of the active pharmaceutical agent (API) can be achieved in the CNS through this pathway bypassing the BBB [9]. 

The following important features of the nasal epithelial should be considered while administering nanoparticles via IN route. The epithelial surface of the nasal mucosa is covered with a protective mucin layer as the primary non-aqueous component of mucus. Mucin is a polymer carrying a complex and heterogeneous structure with domains that undergo a variety of molecular interactions, such as primary hydrophilic/hydrophobic and secondary hydrogen bonds besides electrostatic interactions, which can additionally contribute to the limitation of sufficient nose-to-brain transport of particles due to filtration [10]. In the case of particles that can overcome these hurdles by passing through the mucin layer have a chance for efficient absorption through the nasal mucus. Nasal mucus is a selective barrier with a thickness of 5–15 μm and a pore size of 150 ± 50 nm, which regulates permeability by hindering the transport of particles (>200 nm) across the mucosal epithelia [11]. Those particles, which meet the particle size criterion are able to absorb through different pathways. Extracellular nasal transport can occur across the tight junctions; however, mainly free molecules are able to get over this narrow passage (3.9–8.4 Å). The application of absorption enhancers can facilitate this transport by opening the tight junctions; therefore, the particles with a diameter smaller than 20 nm are more likely to achieve this way of extracellular transport from the nasal cavity to the brain. In the case of intracellular transport, the expected size of the nanoparticles should be smaller than the diameter of the olfactory axons. Previous studies revealed the diameter of the olfactory axons in rabbits is around 200 nm and in humans between 10 and 700 nm. Therefore, the internalization of the nanoparticles is strongly dependent on their concentration, lipophilicity, and particle characteristics [12,13]. 

Liposomes are potential vehicles for nose-to-brain delivery as they consist of a water-soluble core surrounded by a phospholipid membrane, which is able to improve permeation through the nasal mucosa membrane. Therefore, this characteristic increases lipophilicity and facilitates lipophilic molecules crossing the BBB [14]. The encapsulation of the drug into liposomes can reduce its peripheral toxicity and increase bioavailability and CNS retention. Furthermore, it can also preserve the structural stability of the drug. Liposomes are capable of directly penetrating tumor cells by means of endocytosis and thereby release the drugs locally for highly targeted treatment [15]. Previous studies revealed co-encapsulation of antioxidant compounds with drug components into liposomes or niosomes can potentiate the therapeutic effect at the targeting site [16]. Several studies also revealed the synergic potential of nitrosoureas combined with PG, which contributes to reducing the dosage of required alkylating agents [17]. 

Lomustine (LOM) is an alkylating anticancer drug, which non-specifically mutates the cell cycle at the G1 stage, G2 stage, G2/S, and M stage. LOM is able to enhance the sensitivity of tumor cells and induce apoptotic cell death through TRC8-mediated degradation targeting heme oxygenase-1 [18]. Conventionally, LOM is orally administered although it has very low bioavailability and significant side effects. Recent studies revealed *n*-Propyl gallate (PG), an antioxidant compound, also has biological applications including antitumor, anti-angiogenic, and anti-inflammatory effects [19]. PG can induce apoptosis in human leukemia cells and HeLa cells by increasing the reactive oxygen species (ROS) levels or glutathione (GSH) depletion through the activation of caspase-3, 8, and 9 [20]. Therefore, the combination of LOM and PG in a suitable drug carrier can be advantageous, especially through nose-to-brain. By exploiting this alternative route, an increased bioavailability of both LOM and PG is expected at low therapeutic doses reducing the occurrence of side effects [21,22]. Yasaswi et al. reported LOM, loaded nanoparticles, prepared with molecular envelope technology showed improved brain tumor therapy without major toxicity [23]. Yang et al. developed LOM and iohexol (a contrast medium for visualization via Computed Tomography) containing thermo-sensitive liposomes for site-specific delivery into the brain tumor showing increased half-life and bioavailability on the C6 glioma model [24]. Wang et al. also developed LOM-loaded thermo-sensitive liposomes with improved properties for targeted tumor therapy [25].

In this study, LOM and PG were co-encapsulated into liposomes with the aim of developing a suitable carrier system applicable in the treatment of GBM through the intranasal route. The nasal applicability of the novel liposomal formulation was characterized according to nanoparticulate properties (average hydrodynamic diameter, polydispersity index, surface charge, encapsulation efficacy), drug release, and ex vivo nasal diffusion. In vitro cellular uptake and the antiproliferative and cytotoxic effects of liposomes co-encapsulated with PG-LOM on normal cell lines (NIH/3T3 mouse embryonic) and different cancer cell lines (U-87 and U251 glioblastoma as well as A2780 ovarium cancer) were investigated.

## 2. Materials and Methods

### 2.1. Materials

LOM (≥98%), PG (≥98%), and phosphatidylcholine (PCL, ≥99%) from soybean were purchased from Sigma-Aldrich Co. Ltd. (Budapest, Hungary). Cholesterol (CHL, ≥99%), ethanol (96% *v/v*), and sodium chloride physiological solution (0.9% *w/w*) were obtained from Molar Chemicals Ltd., (Budapest, Hungary). All cell lines were purchased from the European Collection of Cell Cultures (Salisbury, UK). All reagents for cell line studies were purchased from Sigma–Aldrich Co. Ltd. (Budapest, Hungary) if not indicated otherwise.

### 2.2. Preparation of Liposomes 

A novel direct pouring method (DPM) was used to develop liposomal formulations. This simple technique was based on a modified solvent evaporation method resulting in the stabilized formulation in the case of applying the optimal composition of lipids. In our previous study, the critical factors of the DPM method were determined by a risk assessment (RA)-based experimental design. The RA is the evaluation of the interdependence between the quality target product profile (QTPP) elements and critical quality attributes (CQAs) and between the critical process parameters (CPPs) and CQAs [26]. The RA was implemented by Lean QbD^®^ software (QbDworks.com, QbDworks LLC., Fremont, CA, USA). Based on the RA, the quantity of PCL, the quantity of CHL, the temperature of evaporation showed the highest severity score, indicating a major influence on QTPP. The optimal ranges of these significant parameters, which were also applied in the present study, were determined using a Box–Behnken experimental design [27]. To reach the optimal lipid composition, CHL and PCL were used in different molar ratios (0.67, 1, 1.33, 1.5, 2, 2.67, 4, and 6 mol/mol for CHL). Lipids were dissolved in 4 mL of ethanol–acetone (3:1) mixture and directly added to 10 mL of purified water. After that, the organic solvents were evaporated at 60 °C under constant stirring (400 rpm) by using a hot-plate magnetic stirrer. Drug loaded liposomes were prepared with the same procedure, during which both PG and LOM were added in different mass ratios (1:1, 1:2, 1:3, 2:1, 3:1, 3:4, and 4:3 *w/w*) to the formulation to reach the optimal composition while dissolving lipids in the organic solvent mixture. Three types of liposomes were prepared containing PG and LOM separately and also combined, namely, PG-loaded liposomes (PG-Lipo), LOM-loaded liposomes (LOM-Lipo), and combined PG- and LOM-loaded Liposomes (PG-LOM-Lipo). After constructing the liposomes, the unencapsulated drug was removed via dialysis at 4 °C by using an 8 kDa MWCO dialysis membrane (Spectra/Por^®^, Spectrum Laboratories Inc., Rancho Dominguez, CA, USA). Liposomal formulations were poured into a dialysis bag and sunk into a beaker containing 500 mL physiological saline solution in order to maintain consistent osmotic pressure. After 24 h dialysis, liposomes were centrifuged for 1.5 h at 13,500 rpm in two cycles, among which the pellet was collected and redispersed in 10 mL of purified water using a vortex mixer at 500 rpm (Biobase MX-S, Jinan, Shandong, China). For solid-state characterization, the redispersed pellet was freeze-dried after a purification process at −40 °C and 0.013 mbar for 12 h using ScanVac CoolSafe 100–9 (LaboGene, ApS, Lynge, Denmark) laboratory apparatus. Secondary drying was carried out at 25 °C and 0.013 mbar for 6 h.

### 2.3. Average Hydrodynamic Diameter, Polydispersity Index, and Surface Charge 

Average hydrodynamic diameter (Z-average), polydispersity index (PDI), and zeta potential of liposomal formulations were analyzed in folded capillary cells using a Malvern Nano ZS instrument (Malvern Instruments, Worcestershire, UK). Measurements were carried out at room temperature (25 °C) with a refractive index of 1.445 in three parallel runs and the average value of runs was evaluated. Our standard acceptable criteria range meeting the requirements of IN delivery for the Z-average, PDI, and zeta potential was set to 100–200 nm, 0.0–0.5, and <−15 or >+15 (mV), respectively [28].

### 2.4. Determination of Encapsulation Efficiency, Loading Capacity, and Drug Content of Compared Formulations 

To measure the encapsulation efficiency (EE) and drug loading capacity (LC), liposomal formulations were centrifuged for 1 h at 4 °C and 16,500 rpm (22,413 RCF) in a Hermle Z323 laboratory centrifuge (Hermle AG, Gossheim, Germany), then, the pellet was purified in two cycles, among which it was redispersed in 1.5 mL of purified water using a vortex mixer (Biobase MX-S, Jinan, Shandong, China). After the purification process, the pellet was collected and dissolved in 1 mL of acetonitrile. The quantification of the PG and LOM was performed by HPLC. Each formulation was measured in triplicate. The encapsulation efficacy of LOM and PG was calculated using the following equation [29]:

(1) $$\mathrm{Encapsulation}\;\mathrm{efficiency}\;\left(\%\right)=\frac{w_{1}}{w_{1}+w_{2}}\times 100$$

where w_1_ represents the amount of liposomal entrapped drug (i.e., recovered from the pellet) and w_2_ represents the amount of freely un-entrapped drug (i.e., detected in the supernatant). 

The LC was calculated via the following equation:

(2) $$\mathrm{Loading}\;\mathrm{capacity}\;\left(\%\right)=\frac{w_{1}-w_{2}}{w_{3}}\times 100$$

where w_1_ represents the initial amount of the drug used for the formulation, w_2_ represents the entrapped drug in the pellet, and w_3_ represents the total amount of lipids applied in the formulation. Drug content (DC) was measured by HPLC after a 10-fold dilution of 1.0 mL liposomal formulation with acetonitrile and filtering through a 0.1 µm membrane filter. 

### 2.5. HPLC Method

The quantification of LOM and PG was carried out via HPLC (Agilent 1260, Agilent Technologies, Santa Clara, CA, USA). In the case of LOM, the stationary phase was a Kinetex^®^ EVO C18 column (150 mm × 4.6 mm, 5 µm (Phenomenex, Torrance, CA, USA). The mobile phases used were Milli-Q^®^ ultrapure water (A) and acetonitrile (B). Twenty microliter samples were analyzed. Separation was performed in two steps by gradient elution at 30 °C. The proportion of starting 50% A eluent was reduced to 25% by 10 min and then raised again to 50% by 11 min. For PG analysis as stationary phase a Gemini-NX^®^ C18 column (150 mm × 4.6 mm, 5 µm (Phenomenex, Torrance, CA, USA) was applied. As the mobile phase, Milli-Q^®^ ultrapure water and acetonitrile in an 80:20 ratio, with an adjusted pH = 3.0 using *ortho*-phosphoric acid. Twenty microliter samples were injected, whereas separation was performed by 10 min isocratic elution at 25 °C temperature with 1 mL/min eluent flow. In both measurements, the eluent flow rate was set to 1 mL/min and the chromatograms were detected at 254 nm using an UV-VIS diode array detector. ChemStation B.04.03 Software (Santa Clara, CA, USA) was used for the evaluation of the data. The linear regression of the calibration was 0.999 in the case of LOM, while it was 0.998 for PG. The quantification limit (LOQ) and detection (LOD) of LOM were 40 ppm and 13 ppm, while in the case of PG, 63 ppm and 21 ppm, respectively.

### 2.6. Differential Scanning Calorimetry (DSC)

DSC measurements were performed using a Mettler Toledo DSC 821e instrument (Mettler–Toledo GmbH, Greifensee, Switzerland) to investigate the thermal behavior of freeze-dried pellets (PG-Lipo, LOM-Lipo, and PG-LOM-Lipo) obtained by centrifugation. Approximately 3–5 mg of liposomal pellets was measured into a 40 µL aluminum pan. As a reference, an empty aluminum pan was used. Both pans were perforated, in order to allow the atmosphere above the sample to expand. Measurement was performed in the temperature range of 25 to 325 °C, at a 10 °C/min heating rate under a constant argon atmosphere with a flow rate of 150 mL/min. Data analysis was performed by using STARe software V9.0 (Mettler–Toledo GmbH, Greifensee, Switzerland). Each measurement was normalized to the sample size, according to the measured weight of each composition.

### 2.7. X-Ray Powder Diffractometry (XRPD)

The XRPD diffractogram of the initial PG, LOM, and freeze-dried pellets obtained by centrifugation (PG-Lipo, LOM-Lipo, and PG-LOM-Lipo) was measured by using a Bruker D8 ADVANCE X-ray powder diffractometer (Bruker AXS GmbH, Karlsruhe, Germany). All the results were obtained using a VANTEC slit detector with Cu K λI radiation (λ = 1.5406). The investigation of all components was carried out at 40 kV and 40 mA in the angular range of 3 to 40° 2θ with a step time of 0.1 s and a step size of 0.007°. The samples were measured on a quartz sample holder at room temperature. The crystallinity index (CI) of both PG and LOM was determined semi-quantitatively based on the total area under diffractograms of the starting compounds (A_crystalline_) compared to the total area under diffractograms of the liposomal formulations (A_crystalline_ + A_amorphous_) [30]:

(3) $$\mathrm{CI}\;\left(\%\right)=\frac{A_{\mathrm{crystalline}}}{A_{\mathrm{crystalline}}+A_{\mathrm{amorphous}}}\times 100$$

### 2.8. In Vitro Release Studies

In vitro release study was performed at 35 °C using dialysis membrane method at 50 rpm constant stirring. 1 mL of liposomal formulations (PG-Lipo, LOM-Lipo, PG-LOM-Lipo, PG suspension, and LOM suspension containing 1-1 mg of PG and LOM, respectively) were poured into an 8 kDa dialysis bag (Spectra/Por^®^, Spectrum Laboratories Inc., Rancho Dominguez, CA, USA) and placed in 50 mL of 7.4 pH PBS. Aliquots were taken at predetermined time intervals for 120 min, then, both LOM and PG contents were determined via the HPLC method, as described above. Each measurement was carried out in triplicate, data were shown as mean ± SD. The in vitro drug release kinetics was evaluated by fitting mathematical models (First order, Zero-order, Korsmeyer–Peppas, Hixson–Crowell, and Higuchi-model) [31].

### 2.9. Stability Studies

The stability of freeze-dried liposomal formulations (PG-Lipo, LOM-Lipo, PG-LOM-Lipo) was evaluated over a period of 4 weeks. Briefly, the formulations were stored at 4 °C and 25 °C (ambient temperature), while samples were withdrawn at specific time intervals (1, 2, 3, and 4 weeks). The samples were analyzed for Z-average, zeta potential, and EE, as described above.

### 2.10. Ex-Vivo Studies

#### 2.10.1. Isolation of Rabbit Nasal Mucosa

Nasal mucosa was isolated from male New Zealand rabbits (body mass 2–3 kg) Immediately after the animals (*n* = 4) were sacrificed by rapid cervical dislocation, nasal cavities of the rabbits were opened surgically, and the nasal mucosa was isolated [32]. The nasal septum was extracted, and the mucosa layers were carefully detached from the septum cartilage by using bone cutters or surgical scissors and cut into 400 ± 50 µm thick slices. This thickness is sufficient to represent the entire mucosal barrier of the nasal cavities, including the epithelial barrier (thickness around 100 µm) as well as part of the underlying connective tissue [33]. Total nasal mucosa was obtained and used for the experiment. To maintain the viability of the excised nasal tissue, it was immediately immersed in ice-cold phosphate buffer saline pH 7.4 for 15 min and aerated (95% O_2_ and 5% CO_2_) until further use [34]. All experiments conformed to the Directive 2010/63/EU of the European Parliament. The protocols have been approved by the Ethical Committee for the Protection of Animals in Research of the University of Szeged, Szeged, Hungary, and by the Department of Animal Health and Food Control of the Ministry of Agriculture and Rural Development (authority approval numbers XIII./4227/2016). 

#### 2.10.2. Ex Vivo Raman Chemical Mapping on Rabbit Nasal Mucosa

Five microliters of PG-Lipo, LOM-Lipo, PG-LOM-Lipo, PG suspension, and LOM suspension containing 1-1 mg of PG and LOM, respectively, were instilled at the epithelial surface of the nasal mucosa. After 60 min, the formulation was wiped off and washed two times with physiological saline solution, and the nasal mucosa was divided into cross-sections into the incision point, then inverted to the cross-sectional side and placed on an aluminum foil-coated glass slide. Ex vivo Raman chemical mapping was performed via a ThermoFisher XDR Dispersive Raman instrument (ThermoFisher Scientific Inc., Waltham, MA, USA) equipped with a CCD camera and a diode laser operating at a wavelength of 780 nm. A 500 µm × 500 µm size surface was analyzed with a step size of 50 µm with an exposure time of 4 s and acquisition time of 4 s, for a total of 4 scans per spectrum in the spectral range of 3500 to 200 cm^−1^ with cosmic-ray and fluorescence corrections. The Raman spectra were normalized, in order to eliminate the intensity deviation between the measured areas.

#### 2.10.3. Ex Vivo Nasal Diffusion Study on Rabbit Nasal Mucosa 

The ex vivo transmucosal passive diffusion study of the formulations under nasal conditions was performed in a modified Side-Bi-Side^®^ type horizontal diffusion apparatus. The diffusion of PG-Lipo, LOM-Lipo, PG-LOM-Lipo, PG suspension, and LOM suspension containing 1-1 mg of PG and LOM, respectively, was tested. The freshly excised rabbit nasal mucosa was mounted between the donor and acceptor compartment (effective diffusion surface area of 0.785 cm^2^). The donor phase consisted of 8.0 mL of Simulated Nasal Electrolyte Solution (SNES, containing 8.77 g/L sodium chloride (NaCl), 2.98 g/L potassium chloride (KCl), 0.59 g/L anhydrous calcium chloride (CaCl_2_) dissolved in purified water, pH 5.6), while the acceptor phase contained 9.0 mL of pH 7.4 PBS. The temperature of both chambers was controlled at 35 ± 0.5 °C using a heating circulator (ThermoHaake C 10-P5, Sigma–Aldrich Co., Ltd., Budapest, Hungary). For the diffusion study, each formulation (1 mL) was placed in the donor compartment of the diffusion cell. Both donor and acceptor compartments were continuously stirred at 100 rpm using magnetic stirrers. Aliquots from the acceptor phase (150 µL) were taken at predetermined time points (1, 3, 5, 10, 15, 30, and 60 min) and replaced with fresh medium. LOM and PG concentrations were determined using HPLC. The flux (*J*) was calculated from the quantity of the drug permeated through the membrane, divided by the surface of the membrane insert (0.785 cm^2^) and the duration of the experiment (µg/cm^2^/h). The permeability coefficient (*K_p_*) was determined from *J* and the drug concentration in the donor phase (*Cd* (µg/cm^3^)). as seen in the following equation:

(4) $$K_{p}\left[\frac{cm}{h}\right]=\frac{J}{C_{d}}$$

Relative permeation at 60 min (RP_60_) was calculated as a quotient of the liposomal formulations compared to the solutions. Statistical analysis was performed via one-way ANOVA with a posthoc Tukey’s comparison test using TIBCO Statistica^®^ 13.4 (Statsoft Hungary, Budapest, Hungary) software.

Change in Z-average and PDI of liposomes after diffusion through the nasal mucosa was also measured in the acceptor phase to predict the fate of the liposomal carrier after passing the nasal mucosa. At the predetermined time points mentioned above, aliquots were withdrawn from the acceptor phase (0.5 mL) and replaced with fresh medium. Z-average and PDI from the aliquots were determined with DLS.

### 2.11. Antiproliferative MTT Assay

The antiproliferative effect of LOM, PG, and their liposome complexes were determined via an in vitro experiment using NIH/3T3 mouse embryonic fibroblasts, A2780 human ovarian cancer, and U87 glioblastoma cells, by means of a MTT ([3-(4,5-dimethylthiazol-2-yl)-2,5-diphenyltetrazolium bromide]) assay. Glioblastoma cells were cultivated in Dulbecco’s Modified Eagle’s Medium supplemented with 1 mM sodium–pyruvate. NIH/3T3 and A2780 cells were cultured in Eagle’s Minimal Essential Medium. Both culture media were supplemented with 10% fetal bovine serum, 1% non-essential amino acids, and an antibiotic–antimycotic mixture. A limited number of human cancer cells and murine fibroblasts (5000/well) were seeded into a 96-well micro-plate and became attached to the bottom of the well overnight. On the second day of the procedure, the test substances were added in serial dilution (applied in six different concentrations). After an incubation period of 72 h, the living cells were assayed by the addition of 20 µL of 5 mg/mL MTT solution. After a 4 h incubation, the medium was removed, and the precipitated formazan was dissolved in 100 µL/well of DMSO during a 60-min period of shaking. Finally, the reduced MTT was assayed at 545 nm, by reading the absorbance, using a Spectrostar Nano spectrophotometer (BMG Labtech, Ortenberg, Germany). Untreated cells were taken as the negative control. All in vitro experiments were carried out on two 96-well microplates with at least five parallel wells in two independent experiments [35].

### 2.12. In Vitro Cellular Uptake Study

In vitro cellular uptake of the liposomal carrier was investigated by propidium iodide (PI) labeled liposomes on U87 and a more resistant U251 glioblastoma cell line. PI-loaded liposomes were prepared with DPM, similarly to PG- and LOM-loaded liposomes using the optimized lipid composition of CHL and PCL, as described previously. The prepared PI-loaded liposomal formulation contained PI in a 50 µg/mL concentration. The cells were seeded into 96-well microplates with 5000 cell/well cell number. All culture media contained 10% fetal bovine serum, antibiotic/antimycotic complex, and non-essential amino acids. The plates were incubated at 37 °C at 5% CO_2_ tension in a humidified atmosphere overnight and the liposomal nanoparticles were added to the wells in order to have 100 μM PI in each well. After a 24h incubation period, the cells were stained with cell track green (CTG) in a 5 μM concentration. The supernatant was removed and 100 μL medium was added to each well. The cells were examined by a Nikon Fluorescent Microscope (Nikon Instruments Inc., Amstelveen, The Netherlands) equipped with a Digital Sight Camera System, including appropriate filters for PI.

### 2.13. Statistical Analysis

The statistical analysis of the results of this research data was performed using TIBCO Statistica^®^ 13.4 (Statsoft Hungary, Hungary). All the results were repeated in triplicate. All data presented are means ± SD. In vitro release and ex-vivo permeability data were processed using one-way analysis of variance (ANOVA). Changes were considered statistically significant at *p* < 0.05. Calculations of the cell-based assay were performed by GraphPad Prism 5 (GraphPad Software; San Diego, CA, USA).

## 3. Results

### 3.1. Optimization of the Liposomal Carrier

**Figure 1:**
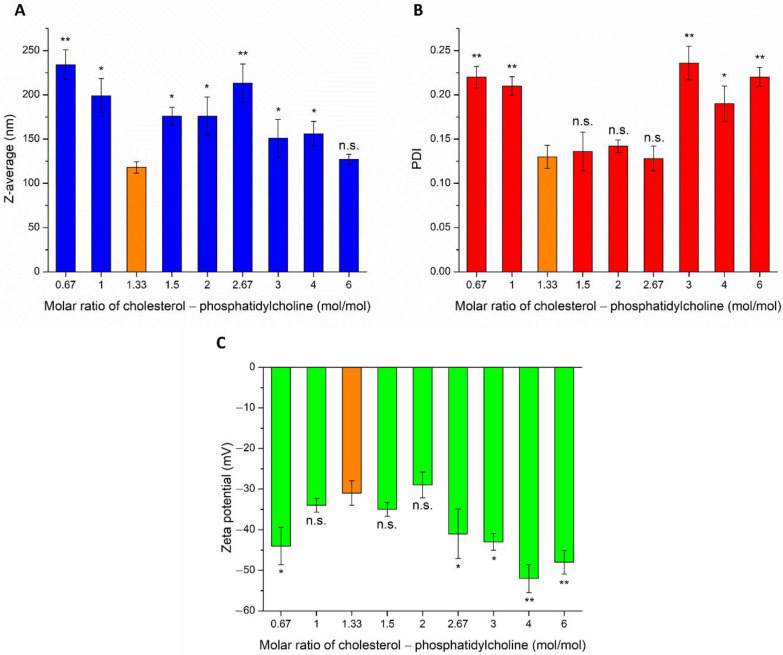
Effect of different molar ratios of cholesterol and phosphatidylcholine (CHL:PCL) for cholesterol on Z-average (**A**), polydispersity index (**B**), and zeta potential (**C**). An ANOVA test was performed to check the significance of the differences between CHL:PCL 1.3 and other compositions, * *p* < 0.05; ** *p* < 0.01; n.s. indicates no significant differences. Measurements were carried out in triplicate (*n* = 3 independent formulations), and data are represented as means ± SD.

The effect of different weight ratios of CHL and PCL were investigated on Z-average, PDI, and zeta potential (ZP), as shown in Figure 1. The particle size of IN administered nanoparticles is required to be reduced as low as possible, to ensure adequate absorption of the encapsulated drug. The Z-average of CHL:PCL 1.33 was found to be significantly lower in comparison to other investigated compositions, the only exception was detected in the case of CHL:PCL 6. From this point of view, these two compositions seemed to be optimal. However, investigating the PDI, it was clearly shown that CHL:PCL 6 had a significantly higher PDI, which predicts hyperdispersed particle size distribution, therefore, uncertain absorption properties and physical stability. The third important parameter was the ZP for defining the optimal composition. From a stability point of view, the higher the absolute value of ZP, the higher the chance to avoid aggregation of nanoparticles as a result of repulsion forces. However, as the mucosal membrane is also negatively charged, the absorption of highly negatively charged nanoparticles may be hindered. This fact also supports the selection of CHL:PCL 1.33 as the optimal composition, showing the least negative surface charge (−31 ± 2.9 mV), which indicates minimal electrostatic repulsion with mucosal membrane, therefore improved nasal absorption. CHL:PCL 1.33 also resulted in one of the lowest Z-average (118 ± 6.3 nm) and narrow PDI (0.13 ± 0.014).

**Figure 2:**
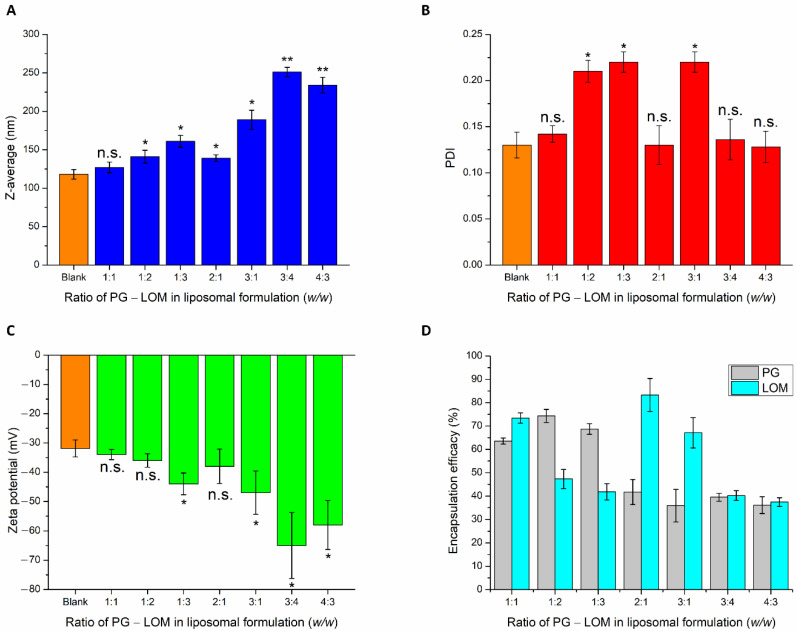
Effect of different PG-LOM ratios on Z-average (**A**), polydispersity index (**B**), zeta potential (**C**), and encapsulation efficacy (**D**) of the optimized liposomal carrier. An ANOVA test was performed to check the significance of the differences between optimized blank liposome and drug-loaded formulation. * *p* < 0.05; ** *p* < 0.01; n.s. indicates no significant differences. Measurements were carried out in triplicate (*n* = 3 independent formulations), and data are represented as means ± SD.

After selecting the optimal lipid composition of the liposomal carrier (CHL:PCL 1.33), drug-loading in different mass ratios was performed. To reach the optimal PG-LOM composition, the effect of different drug ratios on nanoparticulate characteristics, such as Z-average, PDI, zeta potential, and EE, was evaluated (Figure 2). 

PG and LOM tend to encapsulate both inside the phospholipid bilayer and precipitate in a crystalline form in the aqueous vesicle of the liposome, because of their lipophilic nature. This fact is also supported by the slight increase in Z-average while increasing the quantity of the drugs in the formulation. Based on the Z-average values, the applied drug concentrations should be minimized to avoid significant deviation in the vesicle size of the liposomal formulation. The PDI values were not clearly associated with these findings; at higher drug concentrations, similar narrow PDI was observed as in the case of the blank carrier. The ZP values were proportional with increasing drug concentrations, indicating inhibition of aggregation due to electrostatic repulsion. However, increased ZP had a negative effect on the absorption ability through the electrostatic interaction with the negatively charged mucosal membrane. The EE data showed appropriate results in the case of the PG-LOM 1:1 ratio. In the case of other compositions, the EE of one of the drugs was not optimum. All in all, it was revealed that the application of PG-LOM in a 1:1 ratio showed no significant effect on nanoparticle characteristics in comparison to the blank liposomal carrier; therefore, this drug mass ratio was selected for further characterization. The obtained Z-average (127 ± 6.9 nm), PDI (0.142 ± 0.009), ZP (−34 ± 1.7 mV), and high EE (59.87 ± 0.9% of PG, and 71.78 ± 1.5% of LOM, respectively), in the case of both drugs, were suitable for acceptance criteria of nose-to-brain transport [28]. Based on these results, the optimized co-encapsulated formulation (PG-LOM-Lipo) contained both PG and LOM in 1-1 mg/mL after re-dispersion of the pellet in 10 mL purified water, which was applied for further characterizations. For comparison, single drug-loaded liposomes, PG-Lipo, and LOM-Lipo (containing 1-1 mg/mL of PG and LOM, respectively) were also prepared and characterized. The EE, LC, and DC of the optimized and control formulations were determined and compared to each other (Table 1). Each composition resulted in a high EE, LC, and DC, which estimates encapsulated drug found both in the membrane and vesicle of liposomes, indicating controlled drug release from the liposomes mediated by small lipid–drug aggregates or assemblies formed due to the lipophilic nature of drugs. The combined formulation (PG-LOM-Lipo) showed reduced EE and LC in comparison to corresponding single component-loaded liposomes, which can be claimed with the steric hindrance caused by the presence of two drug molecules. However, the relative value of EE and LC of both PG and LOM in PG-LOM-Lipo was quite similar. This effect can probably be explained primarily by the almost same molecular weight of two compounds.

### 3.2. Differential Scanning Calorimetry (DSC) Analysis Results

**Figure 3:**
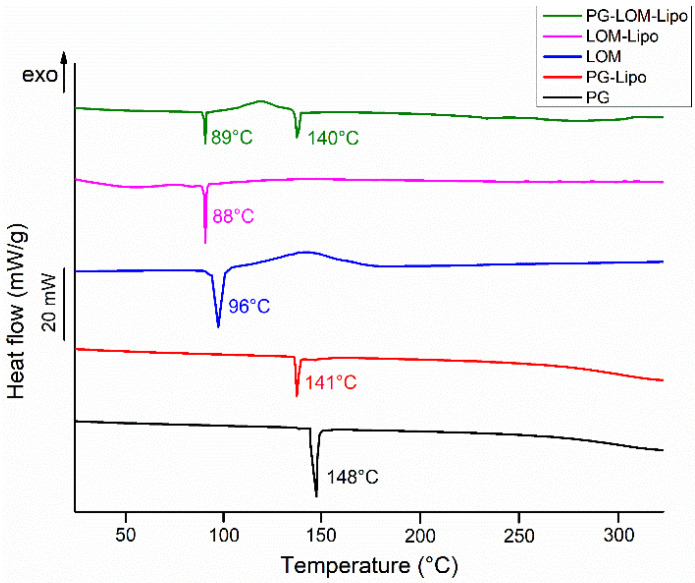
DSC thermogram of liposomal formulations (PG-Lipo, LOM-Lipo, and PG-LOM-Lipo) and initial active ingredients (PG and LOM).

The thermal behavior of pellets (PG-Lipo, LOM-Lipo, and PG-LOM-Lipo) was investigated with DSC (Figure 3). The calorimetric measurement brought supplementary evidence of successful encapsulation of both drugs into the liposomal carrier by the appearance of discrete melting points characteristic of the initial drug. The endothermic peak at 141 °C (PG-Lipo) and 140 °C (PG-LOM-Lipo) can be related to the melting point of PG, while at 88 °C (PG-Lipo) and 89 °C (PG-LOM-Lipo), related to the melting point of LOM. A slight shift in the melting points to lower temperatures can be observed in comparison to initial substances, which might be the effect of the lipids. 

Taking the melting enthalpy values (_∆_*H*) of the thermograms of liposomal formulations into account, a similar tendency in the change in area under endothermic peak was observed in comparison to the DSC curve of initial PG and LOM, which corresponds to the amounts of encapsulated compounds (Table 2) [36]. These findings were in good agreement with the previous EE data (Table 1). No other heat effect was observed in the DSC curves, which indicates no degradation occurred during the liposome preparation process.

### 3.3. XRPD Analysis

**Figure 4:**
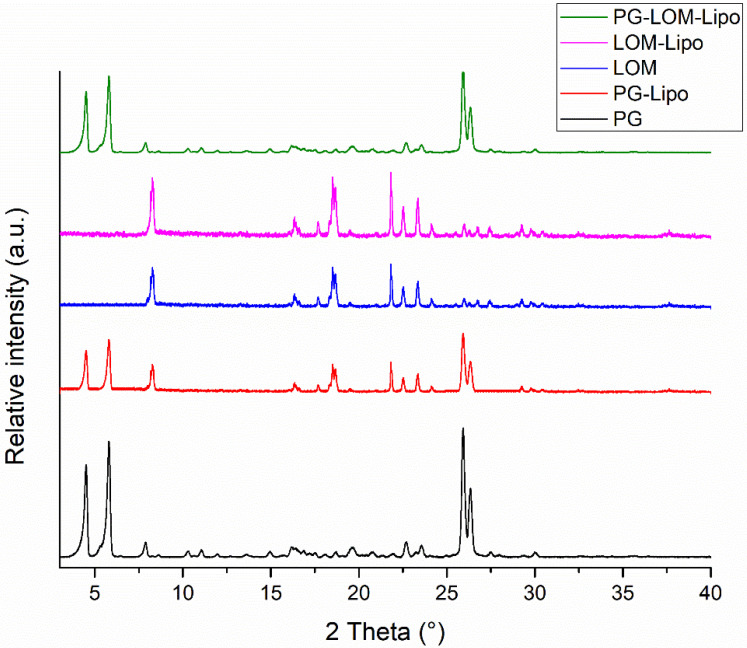
XRPD diffractograms of liposomal formulations and their constituents.

XRPD analysis was carried out to support DSC results. The XRPD diffractograms of liposomal formulations and initial compounds are presented in Figure 4. 

XRPD diffractograms of liposomal pellets supported DSC results. Characteristic peaks related to PG and LOM were present in the liposomal formulations, which supported the crystalline existence of both drugs in the liposomal formulation. The crystallinity index (CI) was calculated in each formulation to compare crystalline fractions of encapsulated drugs (Table 3). 

The CI values clearly indicate that significant PG and LOM can be found in crystalline form in the liposomal formulation, which indicated supplementary evidence of successful co-encapsulation of both PG and LOM.

### 3.4. In Vitro Release Study

**Figure 5:**
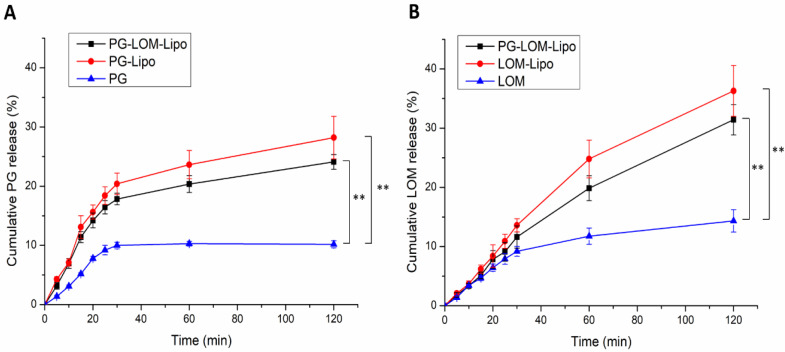
Cumulative drug release of PG- (**A**) and LOM-containing (**B**) formulations in comparison to initial LOM and PG suspension. An ANOVA test was performed to check the significance of the differences between liposomal formulations and initial drug suspension, ** *p* < 0.01. Measurements were performed in triplicate (*n* = 3 independent formulations), and data are represented as means ± SD.

Drug release kinetic of liposomal formulations is a crucial part of the rational design, predicting the subsequent characteristics of a drug delivery system after administration. The in vitro release profile reveals important information on the structure and behavior of the formulation, possible interactions between the drug and lipid composition, and their influence on the rate and mechanism of drug release. The dialysis-based release method is a well-established and useful technique to study in vitro release from nano-particulate delivery systems. Applying a dialysis bag with an 8 kDa cutoff ensured the physical separation of liposomes and suspended particles of the initial control suspension from the release medium and allowed only the passive diffusion of free LOM and PG. The time-dependent in vitro release profiles of LOM- and PG-loaded liposomes were determined with HPLC (Figure 5).

The drug release profile of reference LOM and PG suspension showed only a slight increase; both drugs reached their equilibrium solubility in 60 min. However, in the case of liposomal formulations, a significant increase (~3-fold) in drug release was observed compared to both LOM and PG (**, *p* < 0.01), which can be explained by the tonicity difference in liposomal formulations and release medium. SNES forms a hypotonic environment in comparison to liposomal formulations, which results in swelling of the liposomes, reducing the thickness of the phospholipid bilayer, therefore, enhancing the drug release kinetics of both PG and LOM [29].

LOM release from PG-LOM-Lipo (R^2^ = 0.9971) and LOM-Lipo (R^2^ = 0.9968) followed Hixson–Crowell kinetics, as well as PG release from the PG-LOM-Lipo (R^2^ = 0.994) and PG-Lipo (R^2^ = 0.9947), which is presumably the result of specific surface area and Z-average changes due to drug release and the effect of ion strength on SNES and drug diffusion from liposomes.

### 3.5. Stability Studies

Liposomal formulations are known to suffer several stability issues. Therefore, the development of a stable liposomal formulation is a basic requirement for designing a suitable drug delivery system. The stability of the optimized liposomal formulation—PG-Lipo, PG-LOM-Lipo, and LOM-Lipo—was evaluated in terms of Z-average, surface charge, and EE as a function of the storage period at room temperature (25 °C) and at 4 °C. There was minimal impact on the Z-average and surface charge of liposomes, only a slight increase in particle size (~10 nm), and zeta potential (~7 mV) was found after 4 weeks of storage at 4 °C (Figure S1A,B). However, storing at room temperature (25 °C) for 4 weeks resulted in the aggregation of particles, leading to 199 ± 6.8 nm (PG-Lipo), 202 ± 9.1 nm (PG-LOM-Lipo), and 191 ± 14.2 nm (LOM-Lipo) particle size, which in turn impacted the surface charge, causing a reduction in zeta potential to −7.9 ± 2.6 mV (PG-Lipo), −8.3 ± 1.9 mV (PG-LOM-Lipo), and −11.9 ± 2.5 mV (LOM-Lipo), respectively (Figure S1E,F). The entrapment efficiency of liposomal formulations was found to be stable at 4 °C, with only about a 5–5% drug entrapment reduction after 4 weeks detected for PG and LOM, as seen in Figure S1C,D. However, in the case of storage at 25 °C for 4 weeks, a 25–28% reduction in EE was detected for both PG and LOM (Figure S1G,H), which indicates that for liposomal formulations it is preferable to store them in a cool place to prolong stability.

### 3.6. Ex Vivo Raman Mapping

**Figure 6:**
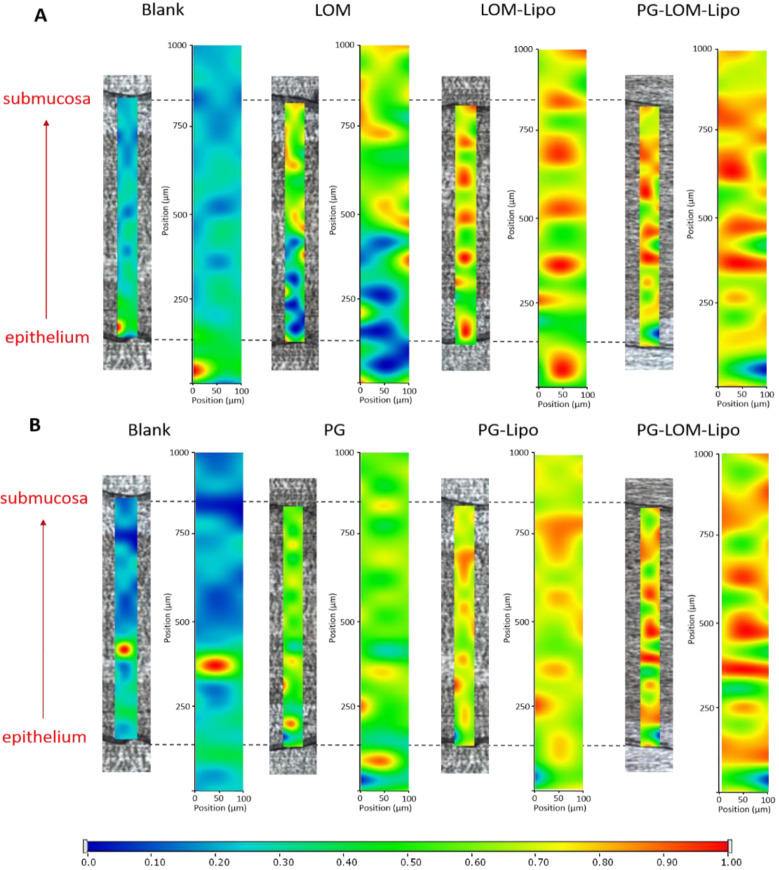
Raman correlation maps of the distribution of LOM (**A**) and PG (**B**) in the rabbit nasal mucosa compared to the non-treated nasal mucosa specimen.

In order to investigate the passive trans-mucosal uptake of liposomal formulations, Raman mapping was applied. Isolated rabbit mucosa was treated with PG-Lipo, LOM-Lipo, PG-LOM-Lipo, PG suspension, and LOM suspension, and the penetration depth was analyzed and compared with non-treated nasal mucosa specimen using Raman correlation mapping. The mucosal distribution correlation maps showed a remarkable Raman intensity on the top of the nasal mucosa specimens in both cases (non-treated, treated), which corresponded to the high protein content of the epithelial layer. Figure 6. shows the Raman maps of treated and non-treated mucosa, profiling the individual spectrum of both LOM (Figure 6A) and PG (Figure 6B). The intensity changes in the Raman maps clearly indicate that both LOM and PG penetrated from the suspension through the nasal mucosa but with less extent in comparison to the encapsulated liposomal carrier. The findings were consistent with the ex vivo diffusion studies results. Based on these results, the improved penetration of both LOM and PG through the nasal mucosa can be supported by using liposomal carriers. 

### 3.7. Ex Vivo Permeation Studies

**Figure 7:**
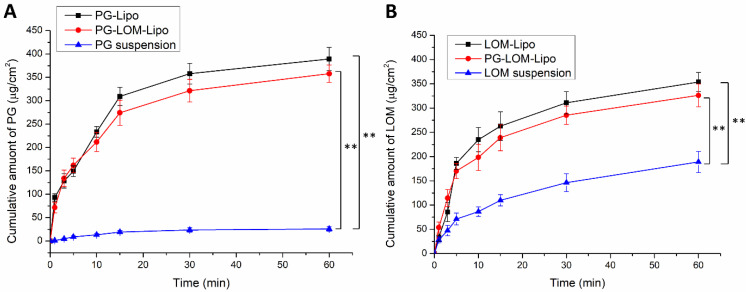
Ex vivo nasal permeability study of PG-containing (**A**) and LOM-containing (**B**) liposomal formulations in comparison to suspensions. An ANOVA test was performed to check the significance of the differences between liposomal formulations and initial drug suspension, ** *p* < 0.01. Measurements were performed in triplicate (*n* = 3 independent formulations), and data are represented as means ± SD.

A modified Side-Bi-Side^®^ type diffusion cell was used for the in vitro nasal permeation study, whereas the diffusion of PG-Lipo, LOM-Lipo, PG-LOM-Lipo was compared to a PG suspension and LOM suspension through rabbit nasal mucosa. Figure 7A shows the cumulative LOM permeation, while Figure 7B, the cumulative PG permeation from donor to acceptor phase through the isolated nasal mucosa. Both in the case of LOM and PG, the permeation was significantly higher in the case of liposomal formulations compared to suspensions. 

Both in the case of PG and LOM liposomal formulations, the flux and permeability coefficient values were significantly higher (** *p* < 0.01) compared to the initial PG and LOM suspensions. Comparing the liposomal carriers, no significant difference in the cumulative permeation of LOM and PG was achieved between LOM-Lipo and PG-LOM-Lipo. The calculated flux, permeability coefficient values, and relative permeability values can be found in Table 4. 

**Figure 8:**
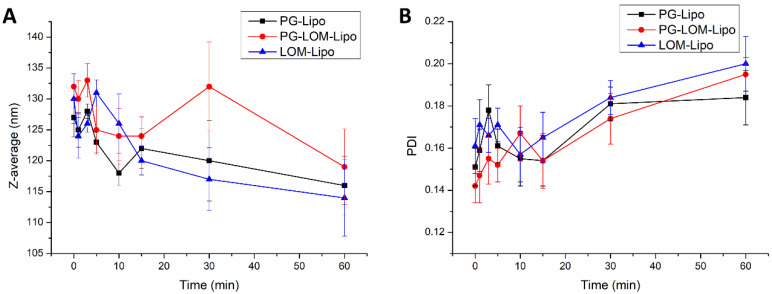
Change in Z-average (**A**) and PDI (**B**) of liposomes during ex vivo permeation study.

The change in vesicle size and PDI in the acceptor phase was also investigated during the ex vivo permeability study. By this measurement, the fate of the liposomal carrier was predicted after nasal uptake. Figure 8 presents the obtained results at the submucosal site of the nasal mucosa.

Each liposomal formulation shows no significant change in the Z-average and PDI in the acceptor compartment after permeation through the nasal mucosa in comparison to the initial Z-average and PDI measured at time point 0 in the donor compartment. These results indicated liposomes may penetrate through the nasal mucosa delivering the encapsulated drug.

### 3.8. Cellular Uptake of Propidium–Iodide Labelled Liposomes

**Figure 9:**
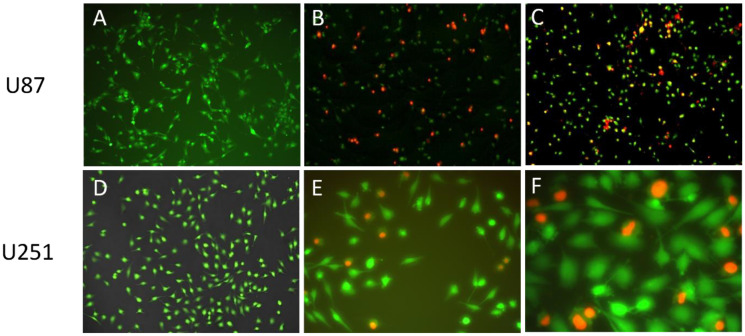
Fluorescence imaging of PI-Lipo cellular uptake on U87 (**B**,**C**) at 10× magnification and U251 cell line at 20× (**E**) and 40× magnification (**F**) after 24 h incubation compared to control group of U87 (**A**) and U251 (**D**) stained with cell track green.

As PI is not membrane permeable, it is a suitable indicator for investigating cellular uptake. By loading PI as a chemical marker in the liposomal carrier, the endocytosis of liposomes can be visualized. Figure 9 shows the fluorescent microscopic results of U87 and U251 cells treated with PI-loaded liposomes (PI-Lipo) for 24 h. 

On both U87 and the more resistant U251 cell lines, successful uptake of liposomes can be detected, as indicated by the red fluorescence inside the cells. These results support that the liposomal carrier was able to transport the encapsulated drug inside the tumor cell, triggering a targeted cytotoxic effect after drug release.

### 3.9. In Vitro Cell Line Studies (Antiproliferative Assay Results)

The antiproliferative effect of LOM-Lipo, PG-Lipo, PG-LOM-Lipo formulations was tested on murine fibroblast cells (NIH), human glioblastoma cell lines (U87), and human ovarian cancer cell lines (A2780) by MTT assays. IC_50_ values of the tested formulations and their 95% confidence intervals (CI) were calculated (Table 5). 

MTT assay results revealed the antioxidant PG can contribute to the anticancer effects by exerting antiproliferative action itself, which can be advantageous in combination with other anticancer drugs, such as LOM. However, in the given experimental environment, PG-Lipo and LOM-Lipo showed higher IC_50_ values on the NIH/3T3 cell line in comparison to PG-DMSO and LOM-DMSO solutions. This phenomenon can be related to the protective effect of the liposomal carrier. From the point of nose-to-brain administration of an anticancer drug, it is essential to reduce the cytotoxic effect on the nasal mucosa. For that reason, liposomes offer a suitable solution. PG-LOM-Lipo showed a lower IC_50_ related to LOM concentrations in comparison to LOM-DMSO, which can be explained by the additional antiproliferative effect of co-encapsulated PG beside LOM. MTT assay results on U87 glioblastoma cell lines showed remarkably higher IC_50_ values of liposomal formulations in comparison to PG-DMSO and LOM-DMSO, which can be attributed to the increased resistance of glioblastoma cells against nanocarrier-mediated drug therapy. However, it is stated that PG and LOM suspension showed poor dissolution and permeability properties both in vitro and ex vivo, which inhibits these formulations from reaching the optimal antiproliferative effect, which was observed in the case of PG-DMSO and LOM-DMSO solutions. To improve tumor targeting through the nasal mucosa, the application of a liposomal carrier is essential, however, due to high EE and LC, a sufficient amount of the drugs can be loaded to provide adequate antiproliferative effects. The cytotoxic effect of formulations was also tested on A2780 ovarium carcinoma cells, which are less resistant than the U87 cell line. It was revealed that the A2780 cell line showed higher sensitivity toward all liposomal formulations. 

## 4. Discussion

Various studies support the in vivo evidence of anticancer and antiangiogenic properties of PG, which is primarily applied as an antioxidant compound [37,38,39]. It has been reported PG can cause apoptosis in human leukemia cells and HeLa cells by inducing reactive oxygen species (ROS) and depleting glutathione [40]. PG also plays a significant role in autophagy and plays a crucial role in cellular physiological processes. 

In our previous studies, the ability of PG loading into two types of lipid nano-carriers (liposomes and solid lipid nanoparticles) was reported. In addition to the high EE of PG-loaded lipid nano-formulation, it was also revealed lipid nano-carriers can preserve the stability of encapsulated drugs, as shown in antioxidant activity measurements. The above-mentioned literature data and our previous findings suggested that the incorporation of PG in combination with an antitumor drug (LOM) in a liposomal carrier can be advantageous in the treatment of GBM via the IN route. The combination of PG and LOM may have an additional benefit, as single compound therapy failed to show meaningful advantages for GBM patients due to poor drug delivery, tumor heterogeneity, and drug resistance mechanisms [41]. Drug combinations, in theory, should take advantage of each compound’s strengths and limitations to improve efficacy and overcome drug resistance. It all begins with the administration method (systemic vs. local), which has a significant impact on these parameters and defines how each component is distributed [42].

Therefore, the present study aimed to optimize and investigate the IN applicability of PG-LOM co-encapsulated liposomes as a suitable formulation for brain tumor therapy. IN drug delivery is a favorable, noninvasive, safe, and effective drug transport method, with the aim of bypassing the BBB. It allows delivering a wide range of therapeutic agents to the brain, including small molecules, growth factors, viral vectors, and even stem cells. Encapsulation of LOM into liposomes provides an interesting idea to resolve the insufficient drug delivery issue to cancer cells and to reduce the side effects on healthy tissues [43,44]. The combination of PG and LOM is novel; their combined anti-proliferative effect has not been studied before. 

First, the composition of the liposomal carrier was optimized. As an optimization criterion, the least negatively surface charge was set, which supports the reduction in electrostatic repulsion with the negatively charged nasal mucosa and also avoids the aggregation of nanoparticles. Based on this requirement, CHL:PCL 1.33 showed the minimal ZP (−31 ± 2.9 mV). This composition also resulted in one of the lowest Z-average (118 ± 6.3 nm) and narrow PDI (0.13 ± 0.014), which is essential to improve drug absorption. After selecting the optimal lipid composition of the liposomal carrier, the appropriate drug ratio was investigated. It was revealed that the application of PG-LOM in 1:1 ratio showed no significant effect on Z-average, PDI, and ZP. Moreover, a high EE was achieved in the case of both drugs, which may facilitate nasal drug absorption. The obtained Z-average (127 ± 6.9 nm), PDI (0.142 ± 0.009), ZP (−34 ± 1.7 mV), and high EE (63.57 ± 1.3% of PG and 73.45 ± 2.2% of LOM, respectively) in the case of both drugs were suitable for acceptance criteria of nose-to-brain transport [28]. Similarly, the DLS and EE results were obtained with co-encapsulated drugs into nanoparticles reported by Yan et al. [45] and are in accordance with the standard acceptance criteria of brain targeted anticancer drug-loaded lipid nanoparticles reported by Khan et al. [46]. Based on these findings, the optimized co-encapsulated formulation PG-LOM-Lipo (CHL:PCL 1.33) contained both PG and LOM in 1 mg/mL. The successful co-encapsulation was further supported by DSC and XRPD measurements. In vitro drug release, permeability studies, and ex vivo Raman measurements showed that the application of liposomes significantly improved the intranasal applicability, predicting increased bioavailability of the co-encapsulated drug components. 

In vitro cellular uptake studies with PI-loaded liposomes supported the drug transport by the liposomal carrier inside the tumor cell, triggering targeted cytotoxic effects after drug release. Antiproliferative studies on fibroblasts and human cancer cells revealed reduced cytotoxicity related to the liposomal carrier. The antiproliferative activity of liposomal formulation was also proved on A2780 ovarium cancer cell lines and U87 glioblastoma cells, however, U87 cells showed increased resistance against liposomal formulations.

The ex vivo study results conducted on both PG- and LOM-loaded liposomal formulations resulted in significantly higher (** *p* < 0.01) flux and permeability coefficients compared to initial PG and LOM suspensions; however, no significant difference in cumulative permeation was obtained between the liposomal formulations. The liposomal formulation showed no significant change in Z-average and PDI after permeation through the nasal mucosa, which indicated that the liposomes may penetrate through the nasal mucosa transporting encapsulated drugs. Praveen et al. investigated the nasal permeability of rhodamine-loaded liposomes prepared similarly from CHL and PCL (Phospholipid90G) [47]. It was revealed that the mucosa treated with liposomes showed high fluorescence intensity due to deeper penetration, while rhodamine solution showed only low penetration. The higher penetration of liposomal formulations through nasal mucosa was explained by the composition, small particle size, and flexibility to change the shape of vesicles, which helped in deeper penetration of the mucosa.

The results obtained indicated the optimized liposomal drug delivery system can be suitable for nose-to-brain delivery of PG and LOM. Guo et al. reported that celecoxib-loaded liposomes consisting of CHL and PCL (Lipoid S100) similar to our composition, improved the brain transport of celecoxib either by penetrating or evading the BBB, upregulating neurogenesis, and targeting Alzheimer’s disease [48].

## 5. Conclusions

In summary, we concluded that all three compared formulations (PG-Lipo, LOM-Lipo, PG-LOM-Lipo) met the acceptance criteria of nose-to-brain administration based on vesicle size, PDI, ZP (mV), and EE (%). In vitro and ex vivo characterizations supported the improved effect of the liposomal carrier on drug release and permeability. In addition to adequate nanoparticulate characteristics, the peripheral side effects of PG and LOM on the nasal mucosa can be reduced due to the application of liposomes by preserving antiproliferative potency at the site of action. The developed and optimized liposomal system provides a suitable tool to treat GBM via the intranasal route.

## Figures and Tables

**Table 1:** Effect of drug components on encapsulation efficacy (EE), loading capacity (LC), and drug content (DC). Data is presented as mean ± SD (*n* = 3 independent formulations).

Liposomal Formulation	EE_PG_ (%)	EE_LOM_ (%)	LC_PG_ (%)	LC_LOM_ (%)	DC_PG_ (%)	DC_LOM_ (%)
PG-Lipo	78.46 ± 3.1	—	19.46 ± 1.1	—	98.56 ± 0.5	—
LOM-Lipo	—	83.48 ± 2.9	—	28.65 ± 1.9	—	97.47 ± 1.6
PG-LOM-Lipo	62.33 ± 1.7	74.11 ± 1.5	12.91 ± 1.8	17.11 ± 2.1	96.24 ± 2.2	96.71 ± 2.9

**Table 2:** Melting points (*T_m_*) and melting enthalpies (_∆_*H*) of encapsulated components.

Liposomal Formulation	*T_m_*_PG_ (°C)	*T_m_*_LOM_ (°C)	_∆_*H*_PG_ (J/g) *	_∆_*H*_LOM_ (J/g) *
PG	148.1	—	111.1	—
LOM	—	96.3	—	140.7
PG-Lipo	141.2	—	86.6	—
LOM-Lipo	—	88.1	—	117
PG-LOM-Lipo	140.2	89.2	59.9	94.4

* g indicates the calculated melting enthalpy values (J) were normalized to sample size.

**Table 3:** Crystallinity index of liposomal formulations.

Liposomal Formulation	*CI_PG_* (%)	*CI_LOM_* (%)
PG-Lipo	68.3	—
LOM-Lipo	—	72.4
PG-LOM-Lipo	43.7	49.1

**Table 4:** Flux (J), permeability coefficient (K_p_), and relative permeability at 60 min (RP_60_) of the liposomal formulations and the reference raw suspensions. * Data are presented as average ± SD (*n* = 3).

Sample	Flux * (µg/cm^2^/h)	K_p_ (cm/h)	RP_60_
LOM values			
LOM suspension	188.918 ± 21.7	0.850	1.00
LOM-Lipo	353.587 ± 19.8	1.591	1.87
PG-LOM-Lipo	326.172 ± 23.7	1.467	1.73
PG values			
PG suspension	25.9 ± 1.7	0.116	1.00
PG-Lipo	399.517 ± 16.7	1.796	15.38
PG-LOM-Lipo	291.236 ± 24.2	1.302	11.46

**Table 5:** IC_50_ values of the tested formulations LOM-Lipo, PG-Lipo, PG-LOM-Lipo, and control solutions of LOM-DMSO and PG-DMSO.

Test Substance	IC_50_ Values (μM) [95% Confidence Interval]		
	NIH/3T3	U87	A2780
LOM-DMSO	264.8 [220.3–318.3]	35.09 [31.02–39.69]	41.88 [35.74–49.09]
PG-DMSO	29.14 [25.10–33.83]	59.42 [51.67–68.32]	14.64 [12.97–16.53]
LOM-Lipo	>500	>500	>100
PG-Lipo	62.93 [56.83–69.69]	275.3 [246.8–307.2]	49.00 [44.24–54.27]
PG-LOM-Lipo *	140.20 [125.3–156.8]	217.60 [190.6–248.4]	70.45 [63.55–78.10]

* IC_50_ values are given as LOM concentration. PG concentrations present at the IC_50_ values of LOM are NIH/3T3: 154.46 μM; U87: 239.74 μM; A2780: 77.62 μM. Blank liposomal samples did not show considerable inhibitory effects.

## Data Availability

The data presented in this study are available on request from the corresponding author.

## Supplementary Materials

The following supporting information can be downloaded at: https://www.mdpi.com/article/10.3390/pharmaceutics14030631/s1, Figure S1: Stability of liposomal formulations evaluated at different temperatures; 4 °C (A–D) and room temperature (25 °C) (E–H) expressed as Z-average (A and E), zeta potential (B and F), and EE regarding to PG (C and G) and LOM (D and H) over period of four weeks.
