# Supplementary material for: Development of Lomustine and n-Propyl Gallate Co-Encapsulated Liposomes for Targeting Glioblastoma Multiforme via Intranasal Administration
Source: Pharmaceutics. 2022 Mar 12;14(3):631. doi: 10.3390/pharmaceutics14030631 (PMC8950329; doi:10.3390/pharmaceutics14030631)
Supplement: Supplementary file 1 [file pharmaceutics-14-00631-s001.zip › pharmaceutics-1615979-supplementary.pdf]

pharmaceutics

Supplementary material: Development of Lomustine and *n*-Propyl Gallate Co-encapsulated Liposomes for Targeting Glioblastoma Multiforme via Intranasal Administration

Gábor Katona [1,*,†], Fakhara Sabir [1,†], Bence Sipos[1], Muhammad Naveed[3], Zsuzsanna Schelz [2], István Zupkó [2] and Ildikó Csóka [1]

[1] Institute of Pharmaceutical Technology and Regulatory Affairs, Faculty of Pharmacy, University of Szeged, Eötvös Str. 6, H-6720 Szeged, Hungary; katona.gabor@szte.hu (G.K.); fakhra.sabir@gmail.com (F.S.); sipos.bence@szte.hu (B.S.); csoka.ildiko@szte.hu (I.C.)
[2] Department of Pharmacodynamics and Biopharmacy, Faculty of Pharmacy, University of Szeged, Eötvös Str. 6, H-6720 Szeged, Hungary; schelz.zsuzsanna@szte.hu (Z.S.) zupko@pharm.u-szeged.hu (I.Z.);
[3] Department of Pharmacology and Pharmacotherapy, Faculty of Medicine, University of Szeged, Dóm sqr. 12; H-6720 Szeged, Hungary; naveed.muhammad@med.u-szeged.hu (M.N.)
* Correspondence: katona.gabor@szte.hu; Tel.: +36-62-545-575
† These authors contribute equally to this paper.

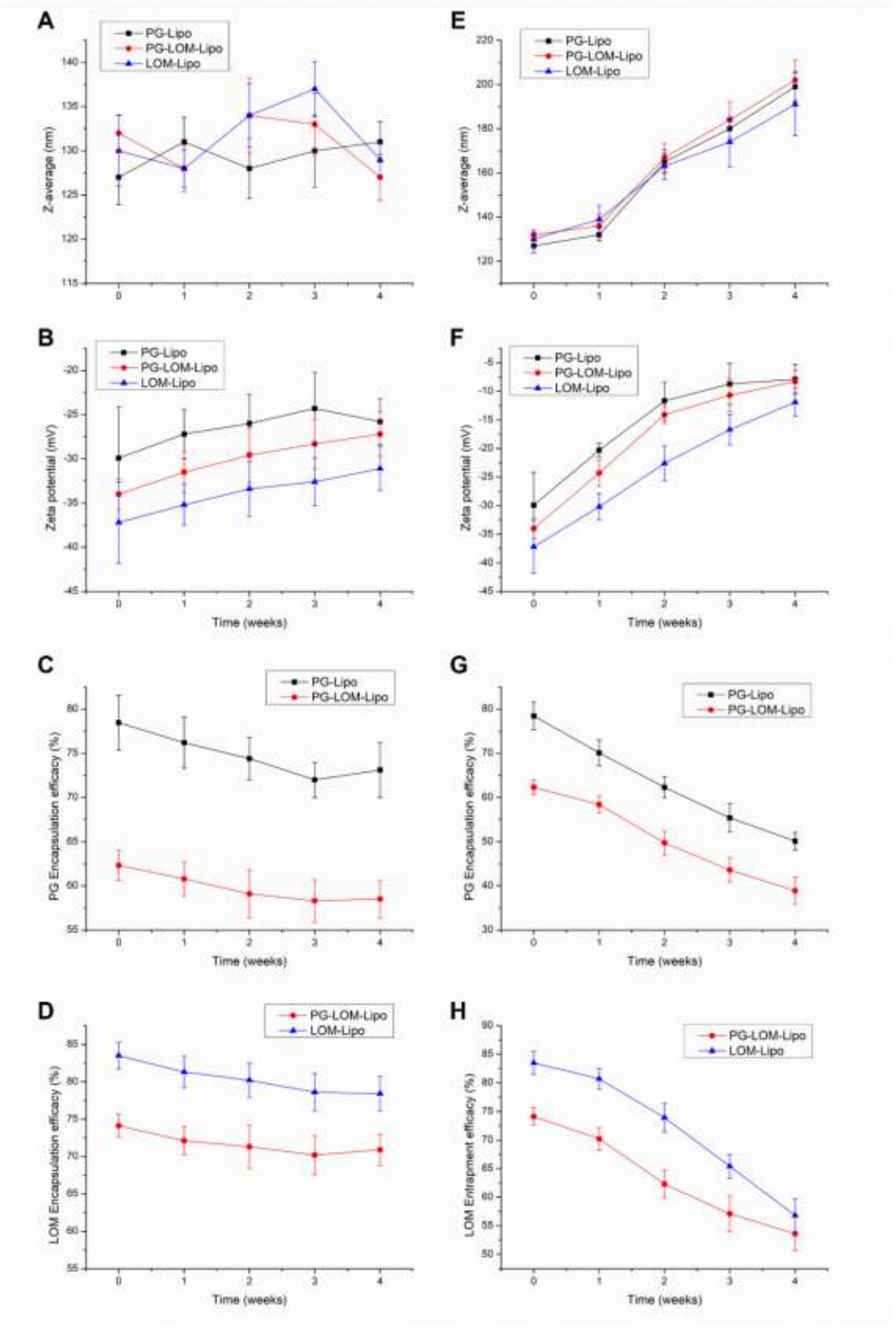

Figure S1. Stability of liposomal formulations evaluated at different temperatures; 4 °C (A-D) and room temperature (25 °C) (E-H) expressed as Z-average (A and E), zeta potential (B and F), and EE regarding to PG (C and G) and LOM (D and H) over period of four weeks.
